# Relationship between Phenolic Compounds and Antioxidant Activity in Berries and Leaves of Raspberry Genotypes and Their Genotyping by SSR Markers

**DOI:** 10.3390/antiox11101961

**Published:** 2022-09-30

**Authors:** Vadim G. Lebedev, Tatyana N. Lebedeva, Elena O. Vidyagina, Vladimir N. Sorokopudov, Anna A. Popova, Konstantin A. Shestibratov

**Affiliations:** 1 Branch of the Shemyakin-Ovchinnikov Institute of Bioorganic Chemistry of the Russian Academy of Sciences, Prospekt Nauki 6, 142290 Pushchino, Russia; 2 Institute of Physicochemical and Biological Problems in Soil Science of the Russian Academy of Sciences, Instituskaya Str. 2, 142290 Pushchino, Russia; 3 All-Russian Scientific Research Institute of Medicinal and Aromatic Plants, Grina Str. 7, 117216 Moscow, Russia; 4 Department of Botany and Plant Physiology, Voronezh State University of Forestry and Technologies Named after G.F. Morozov, Timiryazeva Str. 8, 394087 Voronezh, Russia; 5 Pushchino State Institute of Natural Sciences, Prospekt Nauki 3, 142290 Pushchino, Russia

**Keywords:** antioxidants, anthocyanins, flavonoid biosynthesis, genetic diversity, phenological phase, *Rubus idaeus*, yellow raspberry

## Abstract

The red raspberry is one of the world’s most popular berries. The main direction of its breeding has switched to nutritional quality, and the evaluation of raspberry germplasm for antioxidant content and activity is very important. As berries, raspberry leaves contain valuable bioactive compounds, but the optimal time for their collection is unknown. We evaluated 25 new breeding lines and standard raspberry cultivars for their polyphenolic content and antioxidant capacity. The antioxidant activity of berries correlated better with the content of total phenolics (0.88 and 0.92) and flavonoids (0.76 and 0.88) than with anthocyanins (0.37 and 0.66). Two breeding lines were significantly superior to the standard cultivars and can be used in further breeding. Leaves collected in three phenological phases of the raspberry contained more phenolics (5.4-fold) and flavonoids (4.1-fold) and showed higher antioxidant activities (2.4-fold in FRAP assay, 2.2-fold in ABTS) than berries. The optimal time for harvesting raspberry leaves is the fruit ripening stage, with exceptions for some cultivars. Genetic diversity analysis using microsatellite (SSR) markers from flavonoid biosynthesis genes divided the genotypes into five clusters, generally in agreement with their kinships. The relationship between genetic data based on metabolism-specific SSR markers and the chemical diversity of cultivars was first assessed. The biochemical and genetic results show a strong correlation (0.78). This study is useful for further the improvement of raspberry and other berry crops.

## 1. Introduction

Natural antioxidants, having beneficial effects on human health, have been given considerable attention recently. Berry crops from the genera *Vaccinium*, *Rubus*, *Ribes*, and *Fragaria* are an abundant source of antioxidants of phenolic origin [1,2]. Of them, the red raspberry (*Rubus idaeus* L.) is one of the most popular berries in the world due to its attractive color, excellent taste, and aroma. Interest in this berry is constantly growing. Production of the red raspberry, as well as of its related blackberry and black raspberry, reached 896 thousand tons in 2020, which is 72% more than in 2010 and 2.1 times more than in 2000 [3]. Among berry crops, the raspberry is second only to the blueberry in terms of production increase rate. Russia is the largest raspberry producer, with Poland, Serbia, and the USA constantly among the top five leaders. The raspberry is a rich source of biologically active compounds (phenolics, including anthocyanins and ellagitannins) and nutrients (minerals, vitamins, carotenoids, and organic acids) [4,5]. 

Phenolic compounds are the most common secondary metabolites of plants [6]. They include flavonoids, the most numerous class, as well as phenolic acids, stilbenes, lignans, and coumarins [7]. Polyphenols protect plants from various biotic and abiotic stresses due to their antioxidant abilities based on the stabilization of free radicals [8]. When used in food, polyphenols protect the body from oxidative stress considered to be the common mechanism for the occurrence and progression of the most widespread chronic diseases, thereby contributing to the prevention of cardiovascular, cancer, and inflammatory diseases [9,10]. Of particular interest are anthocyanins which, unlike other flavonoids that are colorless or yellow-colored, are pigment molecules that give attractive colors to flowers and fruits [11]. In edible plants, red, blue, or purple berries are some of the most important sources of anthocyanins [12].

Natural antioxidants are found not only in berries or fruits but in all parts of the plant too. Leaf extracts are currently attracting increasing attention as phytochemicals of nutraceutical importance [13]. Leaves of berry crops have long been used in herbal teas and in traditional medicines. Raspberry leaves are widely used in herbal medicine for treating fever, influenza, diabetes, diarrhea, and colic pain [14]. Experiments have shown that polyphenolic extracts from leaves of *Rubus* spp. have anticancer, antioxidant, antimicrobial, and relaxant properties [15]. Raspberry leaves have been included in the British Pharmacopoeia since 1983, and in 2014, the European Medicines Agency issued a community herbal monograph for the traditional use of red raspberry leaves [16]. Additionally, the red raspberry is one of the most important plants for the preparation of recreational tea [17].

Berries are the main commercial product of the raspberry, strawberry, currant, bilberry, and lingonberry, whereas leaves are considered agro-wastes or by-products [18,19]. However, it has long been shown that blackberry, raspberry, and strawberry leaves have an increased content of phenolics and enhanced antioxidant properties as compared with berries [20]. Subsequent studies have confirmed this for the blueberry [21], cranberry [22], bilberry [23], lingonberry [24], and raspberry [25]. Thus, the leaves can be used as an alternative source of bioactive natural products to develop food additives and nutraceuticals. The biosynthesis of polyphenols and the associated antioxidant activity in various parts of plants are under genetic control, but biotic and abiotic stresses can cause seasonal fluctuations in phenolic content [18,24]. This dependence of the polyphenol concentrations on the time of the year emphasizes the importance of determining the optimal time for collecting plant material when its biological activity is at a maximum.

It is believed that increasing the consumption of raspberry can be a practical strategy for the prevention of a number of chronic human diseases. In recent years the main direction of its breeding has changed from agronomic traits (yield and tolerance to stresses) to fruit quality (sensorial and nutritional) [26]. Knowledge of the genetic diversity in relation to phenolic content in the raspberry germplasm can be used to develop breeding programs by choosing optimal combinations of parents [11]. However, biochemical characteristics alone are not sufficient to confirm genetic diversity. Genotype identification using DNA markers is preferable due to their constancy and reliability because they do not depend on the environment [27]. Microsatellite (simple sequence repeats, SSR) markers are very popular among DNA markers because of their multiallelic nature, codominant inheritance, extensive genome coverage, reproducibility, low cost, and high transferability across species [28,29]. Moreover, these molecular markers can be successfully used in marker-assisted selection to accelerate the creation of new cultivars [30].

SSR markers along with biochemical data have been used to identify true skin color mutants of grapes [31], select parents in potato breeding programs for nutritional quality [32], and prevent the adulteration of olive oil [33]. However, little is known about the relationship between molecular markers and antioxidants. As far as we are aware, the relationships between polyphenol content and genetic diversity have been studied only in the grape [29], cranberry [34], and blueberry [11]. Furthermore, these and other studies have used markers randomly distributed throughout the genome but not associated with specific metabolic pathways. Earlier, we developed a set of SSR markers for structural and regulatory genes of flavonoid biosynthesis. Those markers have been used to compare genetic data with anthocyanin-determined coloration in various species from the genera *Rubus*, *Fragaria*, and *Ribes* [35,36,37].

The purpose of this work is (1) to evaluate the phenolic content and antioxidant activity in berries of new raspberry breeding lines versus the standard cultivars, (2) to compare the phenolic content and antioxidant activity of raspberry leaves when harvested in different phenological phases, and (3) to assess the genetic diversity of raspberry cultivars and breeding lines by SSR markers from flavonoid biosynthesis genes and evaluate their relationship with biochemical data.

## 2. Materials and Methods

### 2.1. Plant Materials

In total, 25 genotypes were used: red raspberry cultivars and breeding lines of Russian origin (lines from Bryansk State Agrarian University), as well as cultivars Polana, Polesie, Polka, Porana Rosa (Poland), Himbo Top (Switzerland), Octavia (UK), and the raspberry × blackberry hybrid Silvan (Australia) (Table 1). All genotypes were propagated in the traditional way, planted in 10-L pots (5 for each genotype) in 2017, and grown under open-air conditions. Berries and leaves were collected from three randomly selected pots (3 replicates) in 2020. Young fully expanded leaves from the upper part of the shoot were collected in the phases of flowering (I), fruit development (II), and fruit ripening (III), which corresponded to stages 65, 77, and 895 of the raspberry phenological scale [38]. Berries were harvested at full maturity at the same time as the last leaf harvest. Leaf and berry samples were frozen immediately upon collection and stored at −80 °C for further use.

Dry matter content of fruits and leaves was determined after drying at 105 °C for 24 h (Table S1).

### 2.2. Preparation of Extracts

In total, 5 g of frozen fruits or 2.5 g of frozen leaf tissue (three replicates for each genotype) were ground with liquid nitrogen using a mortar and then extracted using 15 mL of 80% ethanol in an orbital shaker in the dark for 18 h at room temperature. The supernatant was obtained by centrifugation (4500 rpm, 20 min), and the residue was re-extracted with 6 mL of 80% ethanol in an orbital shaker for 1 h. After centrifugation, supernatant 2 was combined with supernatant 1, and then the combined solution was filled up to 30 mL with 80% ethanol.

### 2.3. Total Phenolic Content (TPC)

Total phenolics were measured using the Folin–Ciocalteau method [39]. In total, 100 µL of the extracts, standards, and blanks were added to 900 µL of the Folin–Ciocalteu reagent (10%) and incubated for 5 min. After incubation, 800 µL of Na_2_CO_3_ (7.5%) was added, mixed, and incubated in the dark for 90 min at room temperature. The absorbance at 760 nm was measured on a Shimadzu UV-1800 spectrophotometer. The calibration curve was plotted using gallic acid solutions at concentrations of 10–300 µg/mL, and the results were expressed in mg of gallic acid equivalents (GAE) per 100 g fresh weight (FW).

### 2.4. Total Flavonoid Content (TFC)

The total flavonoid content was determined by the colorimetric method of Zhishen et al. [40]. Aliquots (200 µL) of extracts or standard solution were added to 600 µL of water and mixed with 60 µL of NaNO_2_ (5%). After 5 min, 60 µL of AlCl_3_ (10%) was added and allowed to stand for 6 min, then 400 µL of 1M NaOH was added to the mixture and the solution was diluted with 480 µL of water. The absorbance was determined at 510 nm. The standard curve was prepared using different concentrations of rutin (50–1000 µg/mL), and the results were expressed as mg rutin equivalents (RE) per 100 g FW.

### 2.5. Total Anthocyanin Content (TAC)

The monomeric anthocyanin content was determined by the pH differential method [41] using two buffers—0.025 M KCl, pH 1.0 and 0.4 M NaAc, pH 4.5. A diluted sample of 0.2 mL was mixed with 1.8 mL of a corresponding buffer and, after a 30 min incubation at room temperature, was read against a blank at 510 and 700 nm. The anthocyanins were calculated as cyanidin-3-glucoside (cyan-3-G) equivalents using an extinction coefficient of 26,900 and molecular weight of 449.2, and the results were expressed as mg per 100 g FW.

### 2.6. ABTS (2,20-Azino-bis (3-ethylbenzothiazoline-6-sulfonic acid) Diamonium Salt) Assay

The antioxidant activity of the extracts against ABTS radical cation was determined according to Re et al. [42]. ABTS°+ stock solution was produced by reacting 7 mM of ABTS and 2.45 mM potassium persulfate after incubation in the dark at room temperature for 16 h. The ABTS°+ working solution was obtained by diluting with ethanol to an absorbance of 0.70 ± 0.02 at 734 nm. Samples were diluted with ethanol so as to give 20–80% inhibition of the blank absorbance. In total, 40 μL of a sample or blank was mixed with 1960 μL of ABTS°+ solution and read at 734 nm after 6 min of incubation in the dark using a Shimadzu UV-1800 spectrophotometer. The calibration curve was plotted using Trolox (0.05–1 mM) as a standard, and the results were expressed as *μ*mol Trolox equivalents (TE) per g FW.

### 2.7. FRAP (Ferric Reducing Antioxidant Power) Assay

The FRAP assay was performed using a colorimetric method by Benzie and Strain [43]. The FRAP reagent was prepared by mixing an acetate buffer (300 mM, pH 3.6), a solution of 10 mM TPTZ in 40 mM HCl, and 20 mM FeCl_3_ at 10:1:1 (*v*/*v*/*v*). The diluted extract (0.2 µL) and FRAP reagent (1.8 µL) were mixed and incubated for 10 min at 37 °C. The absorbance was measured at 593 nm. Trolox was used as a standard for a calibration curve (0.05–1 mM), and the results were expressed as μmol Trolox equivalents (TE) per g FW.

### 2.8. DNA Isolation, PCR Amplification and Fragment Analysis

Total genomic DNA was extracted from young expanding leaves using the STAB method [44]. The quality and quantity of extracted DNA were determined by the NanoDrop 2000 spectrophotometer (Thermo Fisher Scientific Inc., Waltham, MA, USA). The final concentration of each DNA sample was adjusted to 50 ng/μL in TE buffer before the PCR amplification.

For genotyping, the PCR was performed separately for each primer pair using a forward primer labeled with 6-FAM fluorescent dye and an unlabeled reverse primer (Syntol Comp., Moscow, Russia). A total of 11 primer pairs were used: 10 primer pairs were developed from flavonoid biosynthesis genes (RcFH01, FaFS01, FaFS01, RiAS01, FaAR01, RhUF01, RiMY01, RiTT01 [35], FaFH01, RiHL01 [36]), and one pair was developed from an *R. idaeus* Genbank sequence (RiG001, [45]). The PCR amplification was performed in a total volume of 20 μL consisting of 50 ng genomic DNA, 10 pmol labeled forward primer, 10 pmol unlabeled reverse primer, and the PCR Mixture Screenmix (Eurogen JSC, Moscow, Russia). After an initial denaturation at 95 °C for 3 min, DNA was amplified during 33 cycles in a gradient thermal cycler (Bio-Rad Laboratories, Inc., Hercules, CA, USA) programmed for a 30 s denaturation step at 95 °C, a 20 s annealing step at the optimal annealing temperature of the primer pair, and a 35 s extension step at 72 °C. A final extension step was done at 72 °C for 5 min. The PCR generating clear, stable, and specific DNA fragments within an expected length (200–400 bp) were considered successful PCR amplifications.

Separation of amplified DNA fragments was performed in an ABI 3130xl Genetic Analyzer using the S450 LIZ size standard (Syntol Comp., Moscow, Russia). Peak identification and fragment sizing were done using the Gene Mapper v4.0 software (Applied Biosystems, Foster, CA, USA).

### 2.9. Statistical Analysis

The content of phenolic compounds was calculated traditionally: in berries, per 100 g FW; in leaves, per g dry weight (DW). Due to a significant difference in dry matter content in berries and leaves, the content of phenolic compounds in berries for their correct comparison was recalculated per g DW. The antioxidant activity of berries, traditionally measured per g FW, was also recalculated per g DW for comparison with leaves. Data for antioxidant activity, total phenolic, flavonoid, and anthocyanin contents are presented as the mean value ± standard error (SE) of three replications. Statistical analysis was made using Statistica 10 (StatSoft, Tulsa, OK, USA). Significant differences (*p* ≤ 0.05) between the means were evaluated by one-way ANOVA and Duncan’s multiple range test. For comparison of the results of the TPC, TFC, TAC, FRAP, and ABTS assays, the coefficients of correlation were determined by a Pearson correlation test.

Genetic statistics were calculated for polymorphic SSR markers. The number of alleles observed (Ho) and expected (He) heterozygosities, and the value of the polymorphic information content (PIC) for 24 diploid red raspberry cultivars were calculated using the Power Marker 3.25 software [46]. The UPGMA dendrogram was built using data from the Power Marker 3.25 software package and visualized using Tree ViewX Version 0.5.0 software [47]. To test the correlations between the biochemical parameters and the genetic component, the Mantel test was carried out [48]. A correlation was made between a Euclidean matrix of dissimilarity distances generated using averages of biochemical data and a matrix generated for genetic locus size values for each cultivar generated using R v.4.2.1 (https://cran.r-project.org/bin/windows/base/ (accessed on 20 August 2022)).

## 3. Results

### 3.1. Phenolic Content and Antioxidant Activity in Raspberry Berries

The analysis of berries of 14 breeding lines and 10 cultivars of the raspberry, as well as of the hybrid Silvan, showed large variability in their composition (Table 2). By the total phenolic content (TPC), the genotypes differed 2.4 times and 3.1 times by the total flavonoid content (TFC). The TPC and TFC values were at the maximum in the hybrid Silvan (352.7 and 313.7 mg/100 g FW, respectively), but the hybrid was significantly ahead of all raspberry cultivars only in terms of the TFC. A TPC value of more than 300 was noted in 5 lines (a maximum, 349.5 mg GAE/100 g FW, in line 1-45-2) and in only one cultivar (Octavia). By the TFC, six lines exceeded the cultivar Gusar with their maximum content. Lines 1-45-2 and 1-70-1 (261.1 and 235.5 mg RE/100 g FW, respectively) significantly exceeded all raspberry cultivars. The yellow-fruited lines also had higher TPC and TFC than the cultivars. Furthermore, line 9-121-5 was among the leaders by both contents, and the cultivar Porana Rosa had the minimum values among all genotypes.

By the content of anthocyanins, all genotypes were divided into four groups. The total anthocyanin content (TAC) in almost all red-fruited cultivars was from 16.4 to 40.2 mg cyan-3-G/100 g FW, and they differed little from one another. In yellow-fruited cultivars, the TAC was significantly lower (1.1 mg/100 g FW and less). In dark red berries of line 1-70-1, it was 64.9 cyan-3-G/100 g FW—a significantly higher value as compared with all red raspberry genotypes. Finally, in the black-fruited hybrid Silvan, the TAC was 89.8 mg cyan-3-g/100 g FW, which was significantly higher than that of the red raspberry.

The antioxidant activity (AA) of the berries was measured by the ABTS and FRAP assays (Table 3). According to the FRAP assay, three lines with the activities from 33.5 µmol TE/g FW and higher (1-45-2, 2-66-2, and 1-70-1) significantly exceeded all standard cultivars, except the hybrid Silvan with a maximum AA. By the ABTS assay, the AA of the hybrid was slightly lower than that of line 1-45-2 (47.6 and 52.4 µmol TE/g FW, respectively). Of the standard cultivars, the maximum AA using both assays was noted in the new cultivar Karamelka. The yellow-fruited cultivar Porana Rosa showed the lowest AA when measured by both assays. However, while the AA activities of all yellow-fruited cultivars by the FRAP assay were found to be below the average value (25.6 µmol TE/g FW), by the ABTS assay, the activities of the cultivar Abrikosovaya and line 9-121-5 were above the average (34.7 µmol TE/g FW).

The results of assessing the relationship between the contents of phenolic compounds and AA are presented in Table 4. The FRAP assay revealed a very strong (0.921) correlation between the TPC and AA. The correlation between the TFC and AA as measured by the FRAP assay, as well as by the ABTS assay, was slightly lower but still strong (0.755–0.882). The TAC content correlated worse with the AA: by the FRAP assay, the correlation was moderate (0.655), and by the ABTS assay, the correlation was weak (0.373).

### 3.2. Phenolic Content and Antioxidant Activity in Raspberry Leaves

The analysis of raspberry leaves, as well as berries, showed wide variability in the TPC and TFC values (Tables S2 and S3), but we did not succeed in finding anthocyanins in them. The TPC was minimal in the leaves of the cultivar Octavia irrespective of the harvest period (20.7, 27.2, and 31.5 mg GAE/g DW). Leaves of the hybrid Silvan were the richest in total phenolics (about 130 mg GAE/g DW), also regardless of the phenological phase, and significantly exceeded all the red raspberry genotypes (no more than 100 mg GAE/g DW). Among them, depending on the harvest time, high values were demonstrated by the cultivars Porana Rosa, Himbo Top, and lines 1-14-2 and 9-121-4. The minimum amount of flavonoids was also found in the leaves of the cultivar Octavia (from 14.4 to 23.3 mg RE/g DW). The leaders by the TFC were the hybrid Silvan and the cultivar Himbo Top (except stage III), in which their amount varied from 69.9 to 79.6 mg RE/g DW; however, in contrast with its TPC value, the advantage of the hybrid was insignificant. Among the breeding lines, high TFC values were found in lines 2-66-3 (stages I and III) and 9-121-4 (stage II).

The values of the AA differed somewhat from the content of bioactive substances in leaves (Tables S4 and S5). Both techniques showed a minimum activity in the cultivar Octavia at stages II and III but at the flowering stage in the cultivar Polesie (88.4 µmol TE/g DW by the FRAP and 70.1 µmol TE/g DW by the ABTS assay). The maximum activity was noted not in the hybrid, but in the cultivar Himbo Top (stage I, 570.5 and 600.2 µmol TE/g DW), line 9-121-4 (stage II, 646.3 and 836.7 µmol TE/g DW), and in the fruit ripening phase in line 11-110-2 by the FRAP assay (652.9 µmol TE/g DW) and line 2-66-3 by the ABTS assay (841.1 µmol TE/g DW).

Evaluation of the relationship between the content of polyphenols in leaves and their AA value by the FRAP assay showed a very strong correlation between the TFC and the AA in all harvest periods (0.902–0.968; Table 5). The same very strong correlation, as determined by the ABTS assay, was found between the values of TFC and AA, with the exception of stage II (0.819). Both assays showed a weakening of the AA-to-TPC correlation: 0.714 and 0.712 during the flowering period and only a moderate correlation in the subsequent phases (0.595–0.663).

### 3.3. Effect of Phenological Phase on Antioxidant Value of Raspberry Leaves

**Figure 1:**
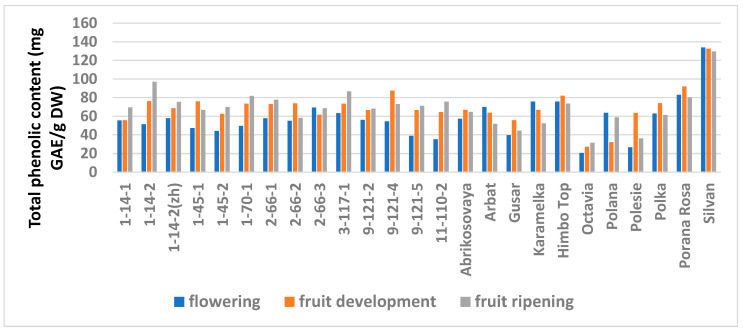
Effects of different harvest periods (phenophase) on total phenolic content in raspberry leaves. Statistical analysis of the data is presented in Table S6.

**Figure 2:**
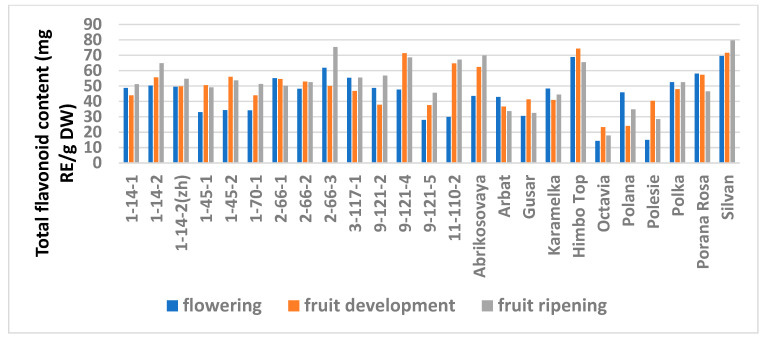
Effect of different harvest periods (phenophase) on total flavonoid content in raspberry leaves. Statistical analysis of the data is presented in Table S6.

**Figure 3:**
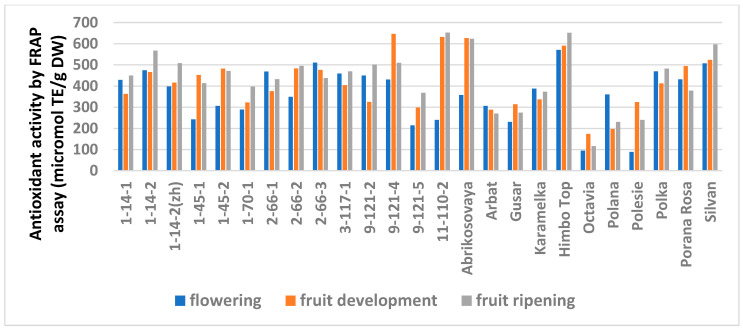
Effect of different harvest periods (phenophase) on antioxidant activity of raspberry leaves measured by FRAP assay. Statistical analysis of the data is presented in Table S7.

**Figure 4:**
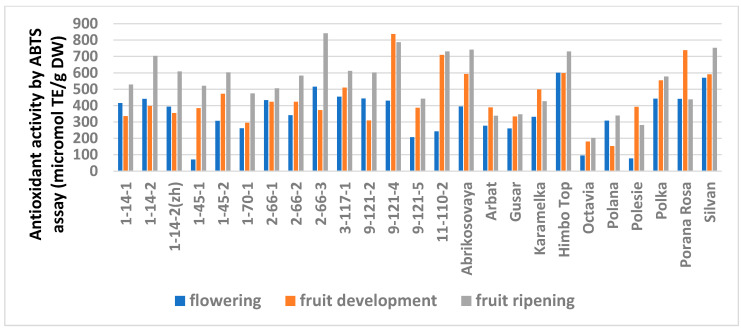
Effect of different harvest periods (phenophase) on antioxidant activity of raspberry leaves measured by ABTS assay. Statistical analysis of the data is presented in Table S7.

The study showed a significant influence of the phenological phases on the values of TPC and TFC (Figure 1 and Figure 2; Table S6), as well as on the AA values (Figure 3 and Figure 4; Table S7) of raspberry leaves. The phenolic content in the leaves was minimal during the flowering period, except for the cultivars Karamelka, Arbat, Himpo Top, Polana, line 2-66-3, and the hybrid Silvan. The statistical analysis confirmed the significance of this difference for seven breeding lines and the cultivar Polesie. The level of the TPC subsequently differed little: only in the cultivars Gusar, Karamelka, and Polesie at stage II was it significantly higher than at stage III. In lines 1-14-2, 11-110-2, and the cultivar Polana, the reverse is the case. The dependence of the TFC on the harvest time was somewhat different: during the flowering period it was minimal only for 13 genotypes (significantly for 7) and for 7 more during the development of fruits, but in all of them the differences were insignificant. Stages II and III also differed little: in line 9-121-5, the content of flavonoids was significantly lower at stage II, and in the cultivars Gusar, Octavia, and Polesie, it was significantly lower at stage III.

Similar trends were observed in the analysis of the AA but with variations depending on the method (Table S6). The FRAP analysis showed no differences for 14 genotypes out of 25. A significantly minimal AA at stage I was observed in six genotypes and a significantly maximal AA was observed at stage III in three (lines 1-14-2, 1-70-1, and 9-121-5). The ABTS assay showed no differences for only six genotypes. The minimum AA at stage I was similar in seven genotypes. However, this method showed a significantly increased activity at stage III for 11 genotypes.

### 3.4. SSR Marker Analysis

A set of 10 SSR markers for flavonoid biosynthesis genes and one marker specific for red raspberry but not blackberry (RiG001) was used for the assessment of the genetic diversity of raspberry genotypes. Four primers were monomorphic (RiTT01, FaAR01, FaFS02, and RiHL01), and the rest were polymorphic. Primers for the RiG001 locus were amplified in the hybrid Silvan. The statistical analysis of SSR loci is summarized in Table 6. In total, 21 alleles were amplified using 7 polymorphic SSR markers. The number of alleles at most loci was two–three, with the exception of the RiMY01 locus which had seven alleles. On average, 3.0 alleles per locus were amplified. The expected heterozygosity (He) ranged from 0.117 (RcFH01) to 0.813 (RiMY01) with an average of 0.287. The observed heterozygosity (Ho) value (percentage of heterozygous individuals among all tested) ranged from 0.125 (RcFH01 and RiAS01) to 0.583 (RiMY01) with a mean value of 0.238. The Ho almost coincided or was lower than expected in most of the examined SSR loci (Table 6). The PIC assayed within loci ranged from 0.110 (RcFH01) to 0.787 (RiMY01) and the mean was 0.265. The RiMY01 locus was the only one whose PIC value exceeded the 0.5 threshold value, indicating the high discriminating capacity of this marker.

**Figure 5:**
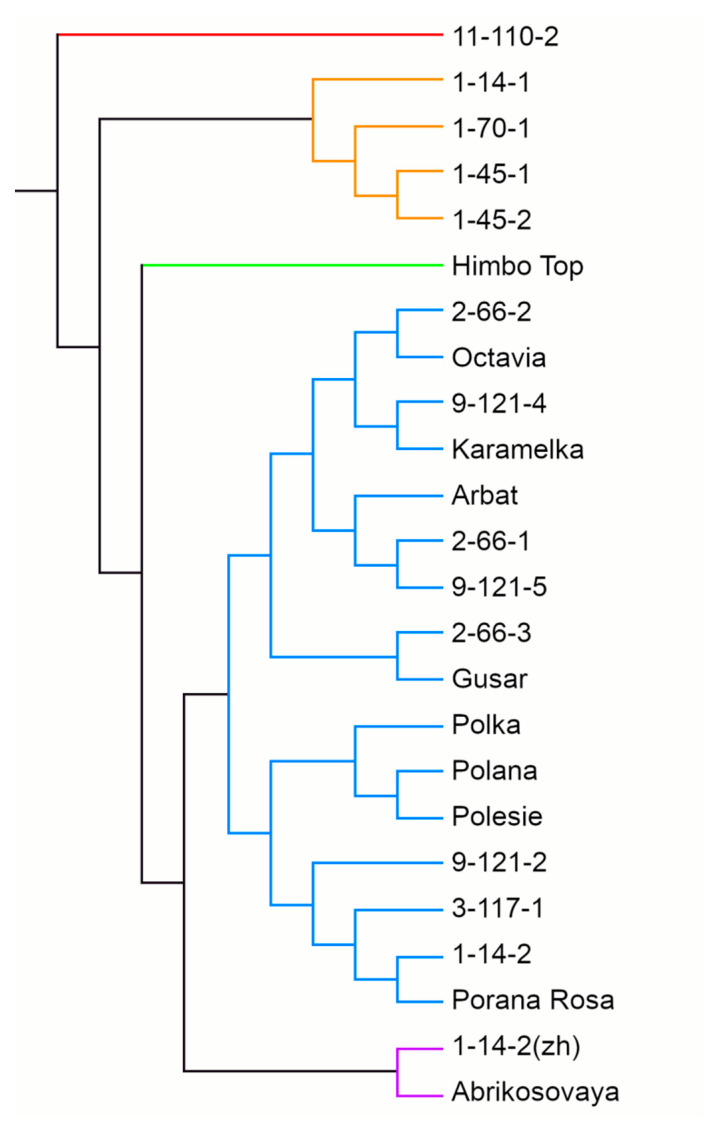
The UPGMA dendrogram of the 24 red raspberry genotypes calculated using SSR markers.

A UPGMA dendrogram based on the results of SSR genotyping grouped raspberry genotypes into five clusters (Figure 5). The largest, cluster 4, included 16 genotypes out of 24, among them 2 yellow-fruited ones, the cultivar Porana Rosa and line 9-121-5. This cluster divides into two subclusters, A and B (of nine and seven genotypes, respectively). The close relationship between three cultivars of Polish breeding—Polka, Polana, and Polesie—should be noted. The yellow-fruited Polish cultivar Porana Rosa also showed similarities to them, getting into the same subcluster 4B. Cluster 2 included four breeding lines—1-14-1, 1-70-1, and two related ones—1-45-1 and 1-45-2. Cluster 5 included two genotypes with yellow berries—the cultivar Abrikosovaya and line 1-14-2(zh). Finally, clusters 1 and 3 included one genotype each: line 11-110-2 and the cultivar Himpo Top of Swiss origin, respectively. This shows their remoteness from other raspberry cultivars.

The correlation between genetic and biochemical data (TPC, TFC, and TAC) according to the Mantel test was high at 0.776.

## 4. Discussion

### 4.1. Impact of Genotype on Polyphenols Content and Antioxidant Capacity of Raspberry Berries

The biochemical composition of berries may depend on various factors, but the genotype is assumed to mostly affect just the content and composition of polyphenols in raspberries [49,50,51]. Due to the great importance of raspberries in Russia, intensive breeding work is being carried out. Considering that the genetic diversity of raspberries with respect to the content of total phenolics and AA is rather limited [26], the evaluation of new breeding lines for these parameters is very important.

In our study, the TPC (148.4–261.1 mg GAE/100 g FW) and TAC (16.4–64.9 mg cyan-3-G/100 g FW) in red raspberry cultivars were lower than those in [49] (278.6–503.9 and 29.2–130.6 mg GAE/100 g FW), but coincided with the results in [50] (183–298 and 18–50 mg GAE/100 g FW) and exceeded the results in [52] for the TPC (59.1–88.8 mg GAE/100 g FW) (all dimensions are the same). Additionally, the dry matter of berries varied from 10.8 to 18.4% (Table S1), which is consistent with the results in [49] (11.9–16.0%). Thus, when growing raspberry plants in pots, the phenolic contents generally coincide with the results of field research.

Yellow-fruited cultivars had much lower TACs (0.3–1.1 mg cyan-3-G/100 g FW), but, for the TPC and TFC, they were among the other red cultivars (Table 2). The low TAC in yellow raspberries (0.5–2 mg cyan-3-G/100 g FW), but the coinciding TPC and TFC compared with red-colored cultivars, has also been reported earlier [5,49,53]. In the yellow cultivar Golden Summit, anthocyanins were not found at all, but by the FRAP-assayed activity, it exceeded the red-colored cultivars [6]. Thus, the yellow-fruited raspberry cultivars, despite their low TAC, also deserve attention as healthy foods.

The hybrid Silvan exceeded the raspberry cultivars in all respects; however, this distinction was significant for the TFC and TAC but insignificant for the TPC. It has also been reported [54] that the hybrids Boysen and Young have similar levels of TPC to the raspberry but contain considerably greater TFC and even greater TAC. The black-colored hybrid Silvan has been shown [5] to significantly exceed, by the TAC, not only the red raspberry cultivars but also the red-colored hybrids tayberry and sunberry. Of all the raspberry genotypes, line 1-45-2 was distinguished by the maximum TPC and TFC, and line 1-70-1 significantly exceeded all the other raspberry genotypes by the TAC. One raspberry breeding line has been found to exceed all standard cultivars by its TFC [53].

Screening of the antioxidant potential of food phenolics is necessarily used when assessing the bioactive value of food products and nutraceuticals [55]. However, one method of analysis cannot give a complete picture of the antioxidant activity of such mixed and complex systems as natural products [56]. We used two methods with the single electron transfer mechanism [8]: the ABTS and FRAP assays. The use of at least two different AA measurement methods makes it possible to obtain objective data [56].

In the raspberry cultivars, the AA varied for both methods of analysis (Table 3). Besides the hybrid Silvan, the maximum AA was observed in lines 1-45-2 and 1-70-1 (FRAP) and 1-45-1 and 1-45-2 (ABTS). Both measurement methods showed a good correlation between the AA and the TPC, a slightly lower correlation with the TFC, and an even lower correlation with the TAC (Table 4). Our data almost coincide with the results of [57] for 12 *Rubus* species, where the correlation of the TAC determined by the ABTS assay was 0.377 and 0.588 by the FRAP assay. Other investigators have also shown that the use of the ABTS and FRAP assays for raspberry and blackberry cultivars yields a stronger correlation between the AA and the TPC (0.89–0.95) and the TFC (0.77–0.90) than with the TAC (0.42–0.67) [5,6,58]. Thus, polyphenols and flavonoids contributed the most, and anthocyanins on a limited scale, to the AA of raspberry fruits. Overall, the correlations with the AA by the FRAP assay were better than by the ABTS. This has already been reported earlier for raspberries [52]. It can be assumed that the FRAP analysis is better suited for the specific substances present in raspberry berries. In general, the breeding lines 1-70-1 and 1-45-2 in terms of the TPC and AA (FRAP) significantly exceeded the standard raspberry cultivars and can be recommended for further use in breeding for nutritional quality. Line 9-101-5 with yellow berries is noteworthy. An increase in the limited genetic diversity of raspberries in terms of phenolic content can be achieved by introducing wild clones into the breeding [26]. In addition, somaclonal variation can also serve as a source of plant material for breeding to enhance antioxidant properties. Increased phenolic content in strawberries was found after cultivation in vitro, and for some plants, the value of estimated genetic gain was enough to start breeding work [59].

### 4.2. Impact of Genotype on Polyphenols Content and Antioxidant Capacity of Raspberry Leaves

The importance of polyphenols as biologically active compounds is generally recognized; they are used as additives in the food, feed, nutraceutical, and pharmaceutical industries [60]. Leaves of berry crops can act as raw materials for the isolation of polyphenols, but only a few studies have shown the antioxidant properties and polyphenol content in raspberry leaves [61].

Our study shows significant differences in the content of phenolics in leaves of various raspberry genotypes (Tables S2 and S3). The TPC in the raspberry differed from 3.1- to 4.0-fold, and the TFC differed from 3.2- to 4.8-fold. The hybrid Silvan significantly exceeded the raspberry cultivars (by 34–61%) irrespective of the harvest time, whereas the TFC was high but did not differ significantly from that of the raspberry. Similar results were obtained for the AA (Tables S4 and S5). Other studies with raspberry and blackberry leaves obtained similar values: the TPC, 47.2–129.2 mg GAE/g DW [19,20,62]. A fourfold difference in the TPC was noted for 26 blackberry species [63] and 70 blueberry genotypes [11], and a twofold difference was found between raspberry and blackberry cultivars [14]. In leaves of 33 strawberry cultivars, the AA determined by the ABTS assay varied 2.5 times [30].

We found no anthocyanins in raspberry leaves. This has been reported earlier [64]. Numerous studies have not shown their presence in the leaves of the bilberry [23], grey-white blueberry [65], lingonberry [18,24], and other berry crops—chokeberry, sea buckthorn, saskatoon, currant, and hawthorn [64]. Reports of the presence of anthocyanins in the leaves of berry crops are rare [19], and their detection can be associated with special extraction conditions.

The correlations of the antioxidant activity with the TPC and TFC for both measurement techniques were similar. A very strong correlation for the TFC measured by the FRAP assay in leaves was observed irrespective of the harvest time and by the ABTS assay at stages I and III (Table 5). The correlations with the TPC were strong and moderate. This shows that phenolics (primarily flavonoids) make a very noticeable contribution to the AA of raspberry leaves. The correlations obtained for the TPC were higher than in other studies of *Rubus* spp. leaves. A correlation of 0.623 with the TPC measured by the ABTS in raspberry leaves has been reported [61]. The correlation between the FRAP and ABTS assays with respect to the TPC in leaves of 26 blackberry species is 0.608 and 0.737, respectively [63]. However, the correlation of the TPC (the FRAP assay) in blueberry leaves has been almost as high, at 0.892 [66]. Additionally, in rabbiteye blueberry leaves, the correlation of the TPC determined by the ABTS assay was also higher than that of the TFC—0.815 and 0.623, respectively [67].

It should be noted that the values of the AA determined by the ABTS assay were always higher than by the FRAP assay both for berries (on average by 37%) and leaves during fruiting (on average by 28%). An approximately 1.5-fold excess of the ABTS over FRAP values (in µmol TE/g FW) has been reported for berries and leaves of various fruit and berry crops [22,63,68].

In terms of dry weight, raspberries contained TPC and TFC on average 5.4 and 4.1 times lower, respectively, than in their leaves harvested simultaneously. Similar results were obtained for bilberry and lingonberry leaves, which had 3.5 and 7 times more total phenolics than berries [23,24]. Raspberry leaves contained about 1.5 times more total phenolics and total flavonoids than fruit pulp [25]. Researchers suggest that such significant differences can be associated with high sugar content in berries, which significantly reduces the proportion of phenolics in them [23,24,69]. The AA in raspberry berries compared with leaves was also lower but to a lesser extent: 2.2 times by the ABTS and 2.4 times by the FRAP assay. Our data coincide with the results of other works, where the AA of raspberry and lingonberry leaves as measured by the FRAP and ABTS assays was 1.5–2 times higher than that of fruits [25,69].

The correlation between raspberry berries and leaves during fruit ripening was weak with respect to the TPC (0.356) and negligible to the TFC (0.138), the AA by the ABTS (0.232), and the AA by the FRAP (0.149). The results obtained for total phenolics were higher than between the leaves and fruits in several fruit and berry species (*r* = 0.03) but lower than for flavonols (*r* = 0.48) [22]. This indicates a low correlation between the leaves and fruits of the plants for the content of polyphenols and AA.

### 4.3. Impact of Harvest Time on Biochemical Parameters of Leaves

The phytochemical composition of leaves depends on the stage of plant growth [66] and environmental conditions [63]. Major research into the phenolic content and AA in raspberry leaves has not evaluated the effect of the harvest season. Such works have been carried out only on various species of *Vaccinium*. To compare the results in all harvest seasons, we selected young leaves at the same stage of development since their age can be of importance. In old blackberry and raspberry leaves (from the lower part of the shoot), the TPC and AA values have been reported to be about two times lower than in young leaves (from the upper part of the shoot) [20].

Analysis showed that the phenophases had no significant effect on the TPC and TFC of leaves in half of all genotypes (Table S5). In a third of the genotypes during the flowering period (I stage), the TPC and TFC were significantly lower than at stages II and III, which did not differ from one another. Occasionally (in two–three genotypes) the TPC and TFC were observed to consistently rise significantly. Our results on the phenolic content are consistent with those for various *Vaccinium* species. In leaves of blueberries picked up at three phenological stages—end of flowering, development of berries, ripening of berries—the phenolic content was maximum at stages II and III irrespective of the ripening earliness of cultivars [70]. The authors explain this by the complete development of leaves at these stages. However, we picked up the leaves in the same phase of their development and assume the cause to be an increase of protection from UV radiation, which increased with increasing daylight hours. It is known that UV-B light can stimulate the synthesis and accumulation of flavonoid compounds that protect the plant from its harmful effects [71,72]. This could correlate with an increase in other metabolic processes since, despite the same phase of development, the dry mass content in leaves consistently increased in all genotypes and averaged 32.7, 36.3, and 39.8% (Table S1). A consistent decrease in water content (from 70 to 34%) in the leaves of two cultivars of highbush blueberry from May to October has been reported [66].

Leaves of the bilberry and lingonberry collected in May, July, and September over two years have also shown a minimum TPC in May, but only for one year out of two, and this in different years for the two berry crops [23,24]. The authors believe that the cause of fluctuations in the biosynthesis of polyphenols can be abiotic stresses caused by contrasting weather conditions over the two years. In our study, the TPC and TFC changed synchronously. This contradicts the results of [67] where the TPC in rabbiteye blueberry leaves collected in May was significantly lower than in September, but the TFC in May was significantly higher than in September.

The AA measured by the FRAP assay, on the whole, correlated with the TPC and TFC, but the ABTS analysis showed a significant excess of the AA at stage III over the previous stages for the genotypes that did not differ in the FRAP analysis (Table S6). Thus, the optimal time for collecting raspberry leaves is the fruit ripening stage, possibly with exceptions for some cultivars.

### 4.4. Genetic Diversity of Raspberry Genotypes

To assess the genetic diversity of raspberry genotypes, we used seven loci for structural flavonoid biosynthesis genes and three for regulatory ones—one for each TF from the MYB-bHLH-WD40 (MBW) ternary protein complex, which regulates flavonoid biosynthesis at the transcriptional level [73]. We also used a pair of primers for the RiG001 locus from *R. idaeus*, which was not amplified in blackberry [45] and black raspberry [35] cultivars. Its specificity for hybrids between the raspberry and blackberry has been controversial. In this work, the RiG001 locus was amplified in the hybrid Silvan, while its amplification was absent in other raspberry × blackberry hybrids—boysenberry, loganberry, tayberry, and Buckingham tayberry [36].

In our work, the average number of alleles, as well as the average values of Ho, He, and PIC, turned out to be lower than in other works where SSR markers from flavonoid biosynthesis genes were used [35,36]. The lower values of diversity parameters are explained by the fact that many closely related breeding lines were used in this work. The RiMY locus showed the highest variability, with seven alleles and PIC = 0.79. It is known that MYB transcription factors (TFs) can enhance or inhibit the synthesis of flavonoids and anthocyanins [54,72]. At the same time, SSR loci from other TFs included in the MBW regulatory complex, RiHL01 (TF bHLH) and RiTT01 (TF WD40), turned out to be monomorphic. This suggests a major role for TF MYB in the regulation of flavonoid biosynthesis.

The dendrogram based on the results of SSR genotyping generally reflects the kinship relationships between the genotypes (Figure 5). For example, the cultivars of the same geographical origin (Polana, Polka, Polesie) were similar, as were the parents and progeny (Karamelka and 9-121-4; 3-117-1 and 9-121-2). The occurrence of related lines in different subclusters, e.g., 2-66-1, 2-66-2, and 2-66-3, is explained by the fact that most breeding lines were obtained by open pollination, i.e., the second parent is unknown.

The yellow-fruited cultivars on the dendrogram divided; Abrikosovaya and 1-14-2(zh) formed a separate cluster, while Porana Rosa and 9-121-5 fell into different subclusters of a large cluster with red-fruited cultivars. Yellow cultivars are usually hybrids or sports of red cultivars. The genetic mechanisms of the formation of yellow coloration in raspberries have not yet been fully studied. Only recently, a 5-bp insertion in the gene of anthocyanidin synthase, which leads to a complete loss-of-function of the enzyme, has been found in the yellow cultivar Anne as compared to the red cultivars [74]. In our study, all yellow cultivars were obtained from different red cultivars, so various mechanisms of the anthocyanin biosynthesis blockage are possible. A separate cluster with Abrikosovaya and 1-14-2(zh) suggests their common mechanism, whereas Porana Rosa and 9-121-5 may have other mechanisms that differ not only from Abrikosovaya and 1-14-2(zh) but also among themselves. We have already noted the separation of yellow-orange raspberry cultivars into different clusters of the dendrogram [35]. The use of highly polymorphic markers for all genes of the flavonoid pathway can clarify the mechanisms of color formation. Additionally, the high variability of MYB TF suggests an important role of expression regulation mechanisms in the formation of yellow coloration.

### 4.5. Comparison of Biochemical and Genetic Data

The Mantel test showed that the TPC, TFC, and TAC in the raspberry strongly correlated with the genetic analysis using specific SSR markers for flavonoid biosynthesis genes (*r* = 0.776). Our previous studies comparing the results of similar SSR genotyping with the color of berries in different species [35,36,37] gave no satisfactory results. We believe the reason is the low resolution of the evaluation of berries by color since cultivars of the same shade can differ significantly in the content of anthocyanins (Table 2). In addition, the evaluation by color does not consider the content of other colorless flavonoids.

There are very few examples of a correlation between chemical and genetic diversity. A comparison of SSR-based data with 18 biochemical parameters of wines found no correlation, and only the comparison with phenylpropanoid molecules gave a strong correlation (Mantel’s *r* = 0.815) [29]. There has been a very good correspondence between the clustering by the SSR analysis and that based on the composition of some fatty acids in olive oil (Mantel’s *r* = 0.801) [75]. On the other hand, no correlation has been found between molecular (ISSR markers) and chemical (essential oils) data in several plant species [29] as well as between SSR markers and starch characteristics in potato tubers [32]. The search for the relationship between biochemistry and genetics in berries of the genus *Vaccinium* has been unsuccessful. Studies have failed to find a correlation between biochemistry and genetics in *Vaccinium* species, cranberry berries [34], or blueberry leaves (Mantel’s *r* = –0.064) [11]. The authors explain the poor correlation between the dispersion of markers across the genome and their location in non-coding regions. This is the main problem with using non-specific genetic markers. It should also be noted that the study in [11] was conducted with leaves that can differ greatly from berries in composition. For example, in bilberry leaves, anthocyanins are absent, whereas in the berries they account for 83–85% of all polyphenols [23]. Significant differences in the qualitative composition of polyphenols between leaves and berries have been also noted for the lingonberry [69].

## 5. Conclusions

The present study evaluated the genetic and biochemical diversity of raspberry germplasm and identified genotypes with a high content of phenolic compounds for use in breeding programs to improve the nutritional quality. Differences between variable colored lines derived from the same raspberry genotype (1-14-1 and 1-14-2 vs. 1-14-2(zh); 9-121-2 and 9-121-4 vs. 9-121-5) can be used to understand the molecular mechanisms underlying color formation in fruits and berries. Assessment of seasonal changes in the content of phenolics and antioxidant activity in raspberry leaves allows for choosing the best harvest period when the leaves have the maximum bioactive value. For the first time, the content of phenolic compounds was compared with genotyping data based on DNA markers from flavonoid biosynthesis genes, which showed a high correlation between biochemical and genetic data. Although the development of specific molecular markers is expensive and time-consuming, they can be more efficient than markers that are randomly distributed across the genome.

## Figures and Tables

**Table 1:** Parentage of tested raspberry cultivars and breeding lines.

Genotype	Fruit Color	Parentage
1-14-1	Red	19-101-20 (open pol.)
1-14-2	Red	19-101-20 (open pol.)
1-14-2(zh)	Yellow	19-101-20 (open pol.)
1-45-1	Red	4-16-1 (open pol.)
1-45-2	Red	4-16-1 (open pol.)
1-70-1	Red	18-183-1 (open pol.)
2-66-1	Red	7-42-5 (open pol.)
2-66-2	Red	7-42-5 (open pol.)
2-66-3	Red	7-42-5 (open pol.)
3-117-1	Red	1-220-1 (open pol.)
9-121-2	Red	3-117-1 × Karamelka
9-121-4	Red	3-117-1 × Karamelka
9-121-5	Yellow	3-117-1 × Karamelka
11-110-2	Red	2-55-10 × Zhar-Ptitsa
Abrikosovaya	yellow-orange	13-222-A (open pol.)
Arbat	Red	821081 × 7518E6
Gusar	Red	Canby × pollen mix
Karamelka	Red	reseeding of seeds
Himbo Top	Red	Autumn Bliss × Rafzeter
Octavia	Red	Glen Ample × EM 5928/114
Polana	Red	Heritage × Zeva Herbsterne
Polesie	Red	86594 × 87432
Polka	red	P89141 (open pol.)
Porana Rosa	yellow	83291 × ORUS 1098-1
Silvan	black	ORUS 742 (Pacific × Boysen) × Marion

**Table 2:** Content of phenolic compounds in berries of raspberry genotypes.

Genotype	TPC (mg GAE/100 g FW)	TFC (mg RE/100 g FW)	TAC (mg cyan-3-G/100 g FW)
1-14-1	236.6 ± 16.3 def	151.2 ± 16.6 fgh	29.2 ± 2.7 def
1-14-2	292.1 ± 20.2 bcd	184.3 ± 12.1 def	39.2 ± 2.7 cd
1-14-2(zh)	267.4 ± 27.9 cde	159.3 ± 18.9 fgh	0.5 ± 0.1 h
1-45-1	305.5 ± 32.5 abc	206.3 ± 25.7 cde	30.2 ± 1.1 cdef
1-45-2	349.5 ± 31.2 ab	261.1 ± 24.4 b	40.2 ± 5.0 c
1-70-1	304.2 ± 14.3 abc	235.5 ± 8.6 bc	64.9 ± 5.9 b
2-66-1	287.0 ± 11.1 cd	194.4 ± 8.3 cdef	39.9 ± 4.9 cd
2-66-2	315.7 ± 40.8 abc	210.4 ± 21.4 cd	35.7 ± 4.8 cde
2-66-3	224.6 ± 3.6 ef	119.8 ± 11.0 hi	31.0 ± 4.8 cdef
3-117-1	224.4 ± 12.3 ef	149.7 ± 10.8 fgh	30.1 ± 0.9 cdef
9-121-2	189.0 ± 14.1 fg	123.7 ± 8.8 ghi	20.9 ± 1.8 fg
9-121-4	215.4 ± 4.1 ef	124.4 ± 3.4 ghi	30.3 ± 4.2 cdef
9-121-5	315.9 ± 16.1 abc	212.3 ± 11.8 cd	1.1 ± 0.1 h
11-110-2	255.4 ± 20.9 cde	173.8 ± 14.2 def	35.0 ± 3.8 cde
Abrikosovaya	225.6 ± 10.7 ef	118.1 ± 7.1 hi	0.7 ± 0.1 h
Arbat	232.5 ± 9.8 def	182.1 ± 8.6 def	39.3 ± 0.8 cd
Gusar	289.6 ± 8.5 cd	189.6 ± 9.1 def	28.3 ± 1.0 ef
Karamelka	274.0 ± 8.8 cde	161.7 ± 4.3 efgh	22.4 ± 3.0 fg
Himbo Top	227.4 ± 21.6 ef	166.1 ± 9.4 defg	31.4 ± 2.7 cdef
Octavia	307.8 ± 15.7 abc	182.7 ± 7.0 def	16.4 ± 1.6 g
Polana	224.2 ± 4.0 ef	166.1 ± 14.2 defg	31.2 ± 1.5 cdef
Polesie	273.9 ± 4.4 cde	187.7 ± 6.3 def	37.3 ± 0.7 cde
Polka	240.2 ± 9.4 def	163.5 ± 3.6 efgh	34.8 ± 1.1 cde
Porana Rosa	148.4 ± 6.0 g	100.5 ± 5.9 i	0.3 ± 0.1 h
Silvan	352.7 ± 17.1 a	313.7 ± 17.2 a	89.8 ± 2.4 a

Results are expressed as mean ± SE (*n* = 3). Different letters represent statistically significant differences (*p* < 0.05).

**Table 3:** Antioxidant activity (micromol TE/g FW) of raspberry berries.

Genotype	FRAP	ABTS
1-14-1	22.9 ± 0.8 de	27.5 ± 1.8 hijk
1-14-2	27.6 ± 2.7 cd	41.2 ± 3.4 bcde
1-14-2(zh)	23.0 ± 1.6 de	33.6 ± 1.6 efghij
1-45-1	30.3 ± 2.4 bc	44.6 ± 4.6 bc
1-45-2	35.9 ± 3.7 ab	52.4 ± 5.4 a
1-70-1	33.5 ± 1.8 ab	35.7 ± 1.6 defgh
2-66-1	26.1 ± 1.2 cde	34.4 ± 1.4 defghi
2-66-2	34.0 ± 4.5 ab	41.1 ± 5.1 bcde
2-66-3	22.8 ± 0.9 de	33.5 ± 0.5 efghij
3-117-1	20.9 ± 1.3 e	26.0 ± 1.4 jkl
9-121-2	14.1 ± 1.2 f	22.7 ± 1.3 kl
9-121-4	23.1 ± 1.1 de	30.6 ± 1.2 fghijk
9-121-5	25.3 ± 1.5 cde	41.2 ± 1.5 bcde
11-110-2	26.5 ± 1.8 cde	38.2 ± 2.7 cdef
Abrikosovaya	22.4 ± 1.5 de	34.8 ± 2.0 defghi
Arbat	23.4 ± 1.4 de	36.5 ± 2.6 cdefg
Gusar	27.9 ± 1.8 cd	34.6 ± 2.5 defghi
Karamelka	28.1 ± 0.7 cd	42.0 ± 1.3 bcd
Himbo Top	22.5 ± 2.2 de	26.9 ± 2.9 ijkl
Octavia	27.2 ± 1.2 cd	36.7 ± 1.2 cdef
Polana	23.9 ± 0.6 de	24.9 ± 0.7 kl
Polesie	25.5 ± 0.5 cde	33.2 ± 1.6 efghij
Polka	22.5 ± 0.6 de	28.4 ± 0.8 ghljk
Porana Rosa	11.7 ± 0.6 f	19.2 ± 0.5 l
Silvan	38.3 ± 1.4 a	47.6 ± 2.7 ab

Results are expressed as mean ± SE (*n* = 3). Different letters represent statistically significant differences (*p* < 0.05).

**Table 4:** Pearson correlations between phenolic compounds and antioxidant activity in raspberry berries.

	FRAP	ABTS
TPC	0.921	0.879
TFC	0.882	0.755
TAC	0.655	0.373

**Table 5:** Pearson correlations between phenolic compounds and antioxidant activity in raspberry leaves.

	Phenophase	FRAP	ABTS
TPC	Flowering	0.712	0.714
	Fruit development	0.595	0.645
	Fruit ripening	0.663	0.642
TFC	Flowering	0.968	0.935
	Fruit development	0.943	0.819
	Fruit ripening	0.902	0.977

**Table 6:** Parameters of genetic variation of red raspberry genotypes.

Locus	Number of Alleles	He	Ho	PIC
RcFH01	2	0.117	0.125	0.110
FaFH01	3	0.190	0.208	0.178
FaFS01	2	0.395	0.292	0.317
RiAS01	2	0.187	0.125	0.169
RhUF01	3	0.156	0.167	0.150
RiMY01	7	0.813	0.583	0.787
RiG001	2	0.153	0.167	0.141
Mean	3.0	0.287	0.238	0.265

## Data Availability

Data is contained within the article and supplementary material.

## Supplementary Materials

The following supporting information can be downloaded at: https://www.mdpi.com/article/10.3390/antiox11101961/s1, Table S1: Dry matter content (%) of raspberry berries and leaves; Table S2: Effect of genotype on total polyphenol content (mg GAE/g DW) in raspberry leaves; Table S3: Effect of genotype on total flavonoid content (mg RE/g DW) in raspberry leaves; Table S4: Effect of genotype on antioxidant activity of raspberry leaves measured by FRAP assay (micromol TE/g DW); Table S5: Effect of genotype on antioxidant activity of raspberry leaves measured by ABTS assay (micromol TE/g DW); Table S6: Effect of different harvest periods (phenophase) on phenolic and flavonoid content in raspberry leaves; Table S7: Effect of different harvest periods (phenophase) on antioxidant activity (micromol TE/g DW) in raspberry leaves.
