# Supplementary material for: Relationship between Phenolic Compounds and Antioxidant Activity in Berries and Leaves of Raspberry Genotypes and Their Genotyping by SSR Markers
Source: Antioxidants (Basel). 2022 Sep 30;11(10):1961. doi: 10.3390/antiox11101961 (PMC9599021; doi:10.3390/antiox11101961)
Supplement: Supplementary file 1 [file antioxidants-11-01961-s001.zip › antioxidants-1927671-supplementary.pdf]

Supplementary Tables

Table S1. Dry matter content (%) of raspberry berries and leaves.

Genotype	Berries	Leaves		
		Flowering	Fruit Development	Fruit Ripening
1-14-1	13.1 ± 0.7	36.8 ± 1.5	38.4 ± 3.0	39.4 ± 2.9
1-14-2	14.4 ± 0.3	35.8 ± 2.2	36.8 ± 1.5	39.1 ± 2.2
1-14-2(zh)	13.7 ± 0.2	35.4 ± 1.7	36.8 ± 2.0	38.8 ± 1.0
1-45-1	15.8 ± 1.2	31.9 ± 2.0	33.1 ± 1.2	37.8 ± 2.1
1-45-2	13.7 ± 0.5	30.1 ± 0.9	36.9 ± 1.9	42.9 ± 3.6
1-70-1	11.9 ± 0.4	34.5 ± 0.7	37.9 ± 2.6	40.7 ± 3.1
2-66-1	14.7 ± 0.5	30.7 ± 0.8	35.7 ± 2.7	39.1 ± 1.2
2-66-2	13.1 ± 1.1	35.0 ± 0.5	38.1 ± 3.2	39.5 ± 2.3
2-66-3	12.9 ± 0.3	29.5 ± 0.8	33.0 ± 2.0	33.3 ± 1.2
3-117-1	15.2 ± 1.6	33.5 ± 0.6	39.7 ± 1.2	41.8 ± 3.0
9-121-2	13.6 ± 1.1	33.9 ± 1.9	39.7 ± 0.7	42.4 ± 1.8
9-121-4	11.4 ± 0.4	30.9 ± 0.9	33.0 ± 1.7	37.8 ± 3.8
9-121-5	16.6 ± 0.6	34.4 ± 2.4	38.0 ± 2.8	40.9 ± 3.6
11-110-2	14.7 ± 0.4	35.8 ± 2.3	36.4 ± 0.9	38.0 ± 2.7
Abrikosovaya	15.1 ± 0.3	34.7 ± 1.3	34.5 ± 1.4	41.5 ± 3.8
Arbat	10.8 ± 0.7	29.7 ± 0.3	36.5 ± 2.2	41.8 ± 1.1
Gusar	17.1 ± 1.3	37.3 ± 1.4	39.0 ± 1.8	40.6 ± 3.4
Karamelka	17.6 ± 1.4	32.7 ± 0.9	35.9 ± 3.1	39.1 ± 0.9
Himbo Top	13.0 ± 0.6	30.1 ± 2.1	35.7 ± 0.5	40.8 ± 2.8
Octavia	14.1 ± 0.8	34.4 ± 2.5	35.3 ± 1.9	37.5 ± 1.0
Polana	15.9 ± 0.1	25.1 ± 0.6	36.5 ± 3.3	39.3 ± 2.4
Polesie	18.4 ± 0.9	27.3 ± 0.8	31.2 ± 1.1	45.7 ± 2.0
Polka	15.9 ± 0.7	31.6 ± 1.9	34.3 ± 2.9	39.2 ± 2.3
Porana Rosa	11.6 ± 0.4	32.1 ± 2.3	35.9 ± 1.2	38.8 ± 1.3
Silvan	12.5 ± 0.7	35.1 ± 0.7	38.6 ± 2.7	40.4 ± 3.4

Table S2. Effect of genotype on total polyphenol content (mg GAE/g DW) in raspberry leaves.

Genotype	Flowering	Fruit Development	Fruit Ripening
1-14-1	55.4 ± 4.2 efg	55.7 ± 3.5 f	69.6 ± 6.4 cdefg
1-14-2	51.5 ± 3.4 efgh	76.1 ± 1.7 bcde	97.0 ± 5.3 b
1-14-2(zh)	57.8 ± 2.9 def	68.6 ± 3.8 cdef	75.5 ± 3.7 cdef
1-45-1	47.2 ± 3.5 fghij	76.0 ± 9.5 bcde	66.8 ± 6.2 defgh
1-45-2	44.2 ± 2.8 ghij	62.4 ± 3.3 def	70.0 ± 5.6 cdef
1-70-1	49.7 ± 3.5 fghi	73.4 ± 6.1 bcdef	81.8 ± 6.5 bcd
2-66-1	57.8 ± 4.8 def	73.0 ± 4.4 bcdef	77.8 ± 2.6 cde
2-66-2	55.1 ± 2.8 efg	73.8 ± 8.2 bcdef	58.3 ± 5.2 fghi
2-66-3	69.4 ± 6.6 cd	61.6 ± 5.5 ef	68.6 ± 3.8 defgh
3-117-1	63.3 ± 5.0 de	73.4 ± 6.9 bcdef	86.7 ± 5.8 bc
9-121-2	56.1 ± 2.6 efg	66.6 ± 3.6 def	68.2 ± 3.4 defgh
9-121-4	54.4 ± 2.6 efg	87.4 ± 3.9 bc	73.1 ± 7.2 cdef
9-121-5	38.8 ± 3.8 ij	66.6 ± 6.0 def	71.1 ± 4.9 cdef
11-110-2	35.2 ± 2.7 jk	64.4 ± 1.0 def	75.7 ± 3.8 cdef
Abrikosovaya	57.3 ± 4.3 def	66.9 ± 6.7 def	64.7 ± 6.7 defgh
Arbat	69.9 ± 3.0 cd	63.8 ± 8.0 def	51.7 ± 5.6 hi
Gusar	39.8 ± 1.4 hij	55.6 ± 1.2 f	44.7 ± 3.0 ij
Karamelka	75.7 ± 3.9 bc	66.8 ± 5.3 def	52.4 ± 2.7 ghi
Himbo Top	75.8 ± 8.2 bc	82.0 ± 6.4 bcd	73.6 ± 6.2 cdef
Octavia	20.7 ± 2.4 l	27.2 ± 1.9 g	31.5 ± 2.8 j
Polana	63.6 ± 2.5 de	32.1 ± 2.4 g	58.9 ± 4.4 fghi
Polesie	26.6 ± 2.7 kl	63.4 ± 3.5 def	36.2 ± 1.4 j
Polka	62.8 ± 2.4 de	74.0 ± 6.7 bcdef	61.4 ± 5.3 efgh
Porana Rosa	82.9 ± 3.8 b	92.0 ± 9.4 b	80.3 ± 5.3 cd
Silvan	133.8 ± 4.0 a	132.5 ± 9.3 a	129.5 ± 8.7 a

Results are expressed as mean ± SE (n = 3). Different letters represent statistically significant differences ($p < 0.05$).

Table S3. Effect of genotype on total flavonoid content (mg RE/g DW) in raspberry leaves.

Genotype	Flowering	Fruit Development	Fruit Ripening
1-14-1	48.8 ± 4.6 def	43.9 ± 4.2 defgh	51.1 ± 1.8 f
1-14-2	50.3 ± 2.3 cdef	55.6 ± 4.1 bcd	64.8 ± 2.8 bcde
1-14-2(zh)	49.5 ± 1.3 def	49.7 ± 2.2 cdefgh	54.7 ± 4.0 def
1-45-1	33.0 ± 1.0 gh	50.6 ± 5.9 cdefg	49.1 ± 2.1 f
1-45-2	34.4 ± 3.8 gh	55.9 ± 1.4 bcd	53.7 ± 3.1 ef
1-70-1	34.2 ± 1.5 gh	43.9 ± 3.4 defgh	51.3 ± 4.8 f
2-66-1	55.1 ± 4.1 cdef	54.6 ± 4.9 bcde	50.2 ± 1.4 f
2-66-2	48.3 ± 2.7 def	52.9 ± 5.5 bcdef	52.5 ± 4.7 ef
2-66-3	61.8 ± 7.0 abc	50.0 ± 2.1 cdefgh	75.4 ± 7.8 ab
3-117-1	55.4 ± 3.3 cde	46.8 ± 4.4 defgh	55.5 ± 3.5 def
9-121-2	48.7 ± 5.6 def	37.8 ± 0.9 gh	56.8 ± 6.6 cdef
9-121-4	47.7 ± 1.8 def	71.3 ± 5.2 a	68.6 ± 4.6 abc
9-121-5	27.9 ± 1.6 h	37.6 ± 1.7 gh	45.6 ± 3.0 fgh
11-110-2	29.9 ± 2.1 h	64.7 ± 5.7 ab	67.1 ± 3.4 abcd
Abrikosovaya	43.5 ± 2.2 efg	62.4 ± 3.7 abc	69.9 ± 5.7 ab
Arbat	42.9 ± 4.3 fg	36.6 ± 1.7 h	33.7 ± 1.8 hij
Gusar	30.6 ± 2.6 h	41.4 ± 1.4 efgh	32.4 ± 1.7 ij
Karamelka	48.3 ± 3.6 def	40.9 ± 4.4 fgh	44.5 ± 0.9 fghi
Himbo Top	68.9 ± 4.1 ab	74.2 ± 8.9 a	65.5 ± 5.5 bcde
Octavia	14.4 ± 1.6 i	23.2 ± 0.9 i	17.9 ± 0.3 k
Polana	45.8 ± 6.2 def	24.1 ± 1.6 i	34.9 ± 3.5 ghij
Polesie	14.9 ± 1.0 i	40.4 ± 2.6 fgh	28.5 ± 1.2 jk
Polka	52.5 ± 5.3 cdef	47.9 ± 4.5 defgh	52.5 ± 1.5 ef
Porana Rosa	58.1 ± 3.0 bcd	57.2 ± 4.3 bcd	46.5 ± 1.9 fg
Silvan	69.5 ± 4.7 a	71.6 ± 3.0 a	79.6 ± 8.1 a

Results are expressed as mean ± SE ($n = 3$). Different letters represent statistically significant differences ($p < 0.05$).

Table S4. Effect of genotype on antioxidant activity of raspberry leaves measured by FRAP assay (micromol TE/g DW).

Genotype	Flowering	Fruit Development	Fruit Ripening
1-14-1	428.9 ± 24.6 bcde	362.5 ± 31.4 fghi	449.3 ± 53.4 defg
1-14-2	474.8 ± 26.1 bc	465.7 ± 27.8 cde	567.3 ± 17.5 abc
1-14-2(zh)	398.4 ± 44.3 cde	416.3 ± 26.4 defg	508.2 ± 40.1 bcde
1-45-1	242.5 ± 28.4 hi	452.4 ± 49.7 cdef	414.0 ± 9.3 defg
1-45-2	306.1 ± 21.7 fgh	482.7 ± 26.0 cd	470.8 ± 41.3 cdefg
1-70-1	289.2 ± 17.3 ghi	322.2 ± 21.5 ghi	397.0 ± 14.8 efg
2-66-1	468.4 ± 38.3 bc	376.1 ± 30.8 efghi	432.6 ± 23.9 defg
2-66-2	349.0 ± 11.9 efg	483.4 ± 45.2 cd	495.3 ± 41.9 bcde
2-66-3	511.0 ± 26.9 ab	476.7 ± 24.0 cd	437.6 ± 31.1 defg
3-117-1	458.9 ± 28.4 bc	403.9 ± 17.9 defgh	469.5 ± 35.7 cdefg
9-121-2	450.0 ± 46.5 bcd	325.1 ± 23.3 ghi	501.4 ± 55.5 bcde
9-121-4	430.7 ± 21.7 bcde	646.3 ± 51.3 a	510.0 ± 20.6 bcd
9-121-5	214.3 ± 9.4 i	298.7 ± 20.0 i	368.4 ± 18.9 ghi
11-110-2	239.9 ± 24.5 hi	631.8 ± 32.9 a	652.9 ± 21.2 a
Abrikosovaya	357.7 ± 30.0 defg	626.7 ± 53.0 a	623.3 ± 58.5 a
Arbat	305.9 ± 33.4 fgh	287.9 ± 32.9 i	270.1 ± 16.4 ij
Gusar	230.7 ± 21.8 hi	314.0 ± 13.1 hi	274.2 ± 28.8 hij
Karamelka	388.2 ± 20.2 cdef	336.9 ± 12.3 ghi	373.5 ± 16.6 fgh
Himbo Top	570.5 ± 32.2 a	590.4 ± 28.8 ab	651.7 ± 25.4 a
Octavia	95.0 ± 11.6 j	174.1 ± 21.0 j	116.3 ± 8.5 k
Polana	360.6 ± 19.0 defg	197.2 ± 15.5 j	230.5 ± 14.2 j
Polesie	88.4 ± 10.9 j	324.5 ± 21.4 ghi	239.6 ± 20.1 j
Polka	469.6 ± 35.1 bc	413.1 ± 39.8 defgh	482.6 ± 56.9 cdef
Porana Rosa	431.9 ± 25.1 bcde	494.0 ± 31.2 cd	378.8 ± 23.9 fg
Silvan	507.4 ± 46.7 ab	523.3 ± 16.7 bc	596.5 ± 46.5 ab

Results are expressed as mean ± SE ($n = 3$). Different letters represent statistically significant differences ($p < 0.05$).

Table S5. Effect of genotype on antioxidant activity of raspberry leaves measured by ABTS assay (micromol TE/g DW).

Genotype	Flowering	Fruit Development	Fruit Ripening
1-14-1	415.5 ± 22.3 bcde	335.1 ± 19.3 ghi	529.1 ± 33.3 def
1-14-2	440.8 ± 35.5 bcd	398.1 ± 26.3 fgh	703.3 ± 64.7 abc
1-14-2(zh)	393.4 ± 19.5 cdef	354.6 ± 28.9 ghi	608.8 ± 57.4 bcd
1-45-1	77.2 ± 10.1 i	383.9 ± 23.1 fghi	520.5 ± 31.5 def
1-45-2	306.7 ± 30.2 fgh	472.0 ± 49.2 def	603.0 ± 73.2 bcd
1-70-1	260.9 ± 19.0 gh	294.7 ± 23.7 i	473.7 ± 35.7 defg
2-66-1	433.6 ± 30.6 bcd	423.4 ± 14.2 efg	505.5 ± 35.6 def
2-66-2	340.7 ± 31.6 defg	423.0 ± 16.7 efg	582.5 ± 68.1 cd
2-66-3	515.0 ± 39.1 ab	372.4 ± 49.1 ghi	841.1 ± 29.9 a
3-117-1	454.2 ± 27.5 bc	509.7 ± 16.2 cde	611.7 ± 27.1 bcd
9-121-2	443.2 ± 42.8 bcd	308.5 ± 16.0 hi	601.7 ± 27.7 bcd
9-121-4	429.3 ± 26.1 bcde	836.7 ± 42.0 a	786.6 ± 61.0 a
9-121-5	207.0 ± 11.6 h	386.5 ± 37.3 fghi	441.6 ± 44.8 efg
11-110-2	242.0 ± 15.0 gh	708.8 ± 17.9 b	730.2 ± 34.4 ab
Abrikosovaya	394.7 ± 17.1 cdef	592.5 ± 12.6 c	741.9 ± 71.4 ab
Arbat	276.3 ± 18.9 gh	388.8 ± 20.9 fghi	337.3 ± 36.9 gh
Gusar	260.6 ± 12.2 gh	333.0 ± 18.0 ghi	346.6 ± 31.2 gh
Karamelka	330.7 ± 38.5 efg	498.6 ± 42.8 de	426.0 ± 33.2 fg
Himbo Top	600.2 ± 64.0 a	598.9 ± 12.0 c	729.8 ± 36.8 ab
Octavia	95.4 ± 6.6 i	180.0 ± 7.6 j	202.3 ± 11.6 i
Polana	307.6 ± 31.4 fgh	152.1 ± 8.0 j	339.4 ± 8.7 gh
Polesie	70.1 ± 5.4 i	392.6 ± 20.4 fgh	281.0 ± 25.1 hi
Polka	441.8 ± 30.8 bcd	554.5 ± 34.3 cd	576.8 ± 41.5 cde
Porana Rosa	440.4 ± 40.8 bcd	738.4 ± 60.4 b	437.3 ± 48.3 efg
Silvan	569.2 ± 63.5 a	590.7 ± 21.1 c	752.5 ± 21.8 a

Results are expressed as mean ± SE ($n = 3$). Different letters represent statistically significant differences ($p < 0.05$).

Table S6. Effect of different harvest periods (phenophase) on phenolic and flavonoid content in raspberry leaves.

Genotype	Phenophase	TPC (mg GAE/g DW)	TFC (mg RE/g DW)
1-14-1	I	55.4 ± 4.2	48.8 ± 4.6
	II	55.7 ± 3.5	43.9 ± 4.2
	III	69.6 ± 6.4	51.1 ± 1.8
1-14-2	I	51.5 ± 3.4 c	50.3 ± 2.3 b
	II	76.1 ± 1.7 b	55.6 ± 4.1 ab
	III	97.0 ± 5.3 a	64.8 ± 2.8 a
1-14-2(zh)	I	57.8 ± 2.9 b	49.5 ± 1.3
	II	68.6 ± 3.8 ab	49.7 ± 2.2
	III	75.5 ± 3.7 a	54.7 ± 4.0
1-45-1	I	47.2 ± 3.5	33.0 ± 1.0 b
	II	76.0 ± 9.5	50.6 ± 5.9 a
	III	66.8 ± 6.2	49.1 ± 2.1 a
1-45-2	I	44.2 ± 2.8 b	34.4 ± 3.8 b
	II	62.4 ± 3.3 a	55.9 ± 1.4 a
	III	70.0 ± 5.6 a	53.7 ± 3.1 a
1-70-1	I	49.7 ± 3.5 b	34.2 ± 1.5 b
	II	73.4 ± 6.1 a	43.9 ± 3.4 ab
	III	81.8 ± 6.5 a	51.3 ± 4.8 b
2-66-1	I	57.8 ± 4.8 b	55.1 ± 4.1
	II	73.0 ± 4.4 a	54.6 ± 4.9
	III	77.8 ± 2.6 a	50.2 ± 1.4
2-66-2	I	55.1 ± 2.8	48.3 ± 2.7
	II	73.8 ± 8.2	52.9 ± 5.5
	III	58.3 ± 5.2	52.5 ± 4.7
2-66-3	I	69.4 ± 6.6	61.8 ± 7.0
	II	61.6 ± 5.5	50.0 ± 2.1
	III	68.6 ± 3.8	75.4 ± 7.8
3-117-1	I	63.3 ± 5.0	55.4 ± 3.3
	II	73.4 ± 6.9	46.8 ± 4.4
	III	86.7 ± 5.8	55.5 ± 3.5
9-121-2	I	56.1 ± 2.6	48.7 ± 5.6
	II	66.6 ± 3.6	37.8 ± 0.9

	III	68.2 ± 3.4	56.8 ± 6.6
9-121-4	I	54.4 ± 2.6 b	47.7 ± 1.8 b
	II	87.4 ± 3.9 a	71.3 ± 5.2 a
	III	73.1 ± 7.2 a	68.6 ± 4.6 a
9-121-5	I	38.8 ± 3.8 b	27.9 ± 1.6 c
	II	66.6 ± 6.0 a	37.6 ± 1.7 b
	III	71.1 ± 4.9 a	45.6 ± 3.0 a
11-110-2	I	35.2 ± 2.7 c	29.9 ± 2.1 b
	II	64.4 ± 1.0 b	64.7 ± 5.7 a
	III	75.7 ± 3.8 a	67.1 ± 3.4 a
Abrikosovaya	I	57.3 ± 4.3	43.5 ± 2.2 b
	II	66.9 ± 6.7	62.4 ± 3.7 a
	III	64.7 ± 6.7	69.9 ± 5.7 a
Arbat	I	69.9 ± 3.0	42.9 ± 4.3
	II	63.8 ± 8.0	36.6 ± 1.7
	III	51.7 ± 5.6	33.7 ± 1.8
Gusar	I	39.8 ± 1.4 b	30.6 ± 2.6 b
	II	55.6 ± 1.2 a	41.4 ± 1.4 a
	III	44.7 ± 3.0 b	32.4 ± 1.7 b
Karamelka	I	75.7 ± 3.9 a	48.3 ± 3.6
	II	66.8 ± 5.3 a	40.9 ± 4.4
	III	52.4 ± 2.7 b	44.5 ± 0.9
Himbo Top	I	75.8 ± 8.2	68.9 ± 4.1
	II	82.0 ± 6.4	74.2 ± 8.9
	III	73.6 ± 6.2	65.5 ± 5.5
Octavia	I	20.7 ± 2.4 b	14.4 ± 1.6 b
	II	27.2 ± 1.9 ab	23.2 ± 0.9 a
	III	31.5 ± 2.8 a	17.9 ± 0.3 b
Polana	I	63.6 ± 2.5 a	45.8 ± 6.2 a
	II	32.1 ± 2.4 b	24.1 ± 1.6 b
	III	58.9 ± 4.4 a	34.9 ± 3.5 ab
Polesie	I	26.6 ± 2.7 c	14.9 ± 1.0 c
	II	63.4 ± 3.5 a	40.4 ± 2.6 a
	III	36.2 ± 1.4 b	28.5 ± 1.2 b
Polka	I	62.8 ± 2.4	52.5 ± 5.3
	II	74.0 ± 6.7	47.9 ± 4.5
	III	61.4 ± 5.3	52.5 ± 1.5
Porana Rosa	I	82.9 ± 3.8	58.1 ± 3.0
	II	92.0 ± 9.4	57.2 ± 4.3
	III	80.3 ± 5.3	46.5 ± 1.9
Silvan	I	133.8 ± 4.0	69.5 ± 4.7
	II	132.5 ± 9.3	71.6 ± 3.0
	III	129.5 ± 8.7	79.6 ± 8.1

Results are expressed as mean ± SE ($n = 3$). Different letters represent statistically significant differences ($p < 0.05$).

Table S7. Effect of different harvest periods (phenophase) on antioxidant activity (micromol TE/g DW) in raspberry leaves.

Genotype	Phenophase	FRAP	ABTS
1-14-1	I	428.9 ± 24.6	415.5 ± 22.3 b
	II	362.5 ± 31.4	335.1 ± 19.3 b
	III	449.3 ± 53.4	529.1 ± 33.3 a
1-14-2	I	474.8 ± 26.1 b	440.8 ± 35.5 b
	II	465.7 ± 27.8 b	398.1 ± 26.3 b
	III	567.3 ± 17.5 a	703.3 ± 64.7 a
1-14-2(zh)	I	398.4 ± 44.3	393.4 ± 19.5 b
	II	416.3 ± 26.4	354.6 ± 28.9 b
	III	508.2 ± 40.1	608.8 ± 57.4 a
1-45-1	I	242.5 ± 28.4 b	77.2 ± 10.1 c
	II	452.4 ± 49.7 a	383.9 ± 23.1 b
	III	414.0 ± 9.3 a	520.5 ± 31.5 a
1-45-2	I	306.1 ± 21.7 b	306.7 ± 30.2 b
	II	482.7 ± 26.0 a	472.0 ± 49.2 ab

	III	470.8 ± 41.3 a	603.0 ± 73.2 a
1-70-1	I	289.2 ± 17.3 b	260.9 ± 19.0 b
	II	322.2 ± 21.5 b	294.7 ± 23.7 b
	III	397.0 ± 14.8 a	473.7 ± 35.7 a
2-66-1	I	468.4 ± 38.3	433.6 ± 30.6
	II	376.1 ± 30.8	423.4 ± 14.2
	III	432.6 ± 23.9	505.5 ± 35.6
2-66-2	I	349.0 ± 11.9	340.7 ± 31.6 b
	II	483.4 ± 45.2	423.0 ± 16.7 b
	III	495.3 ± 41.9	582.5 ± 68.1 a
2-66-3	I	511.0 ± 26.9	515.0 ± 39.1 b
	II	476.7 ± 24.0	372.4 ± 49.1 c
	III	437.6 ± 31.1	841.1 ± 29.9 a
3-117-1	I	458.9 ± 28.4	454.2 ± 27.5 b
	II	403.9 ± 17.9	509.7 ± 16.2 b
	III	469.5 ± 35.7	611.7 ± 27.1 a
9-121-2	I	450.0 ± 46.5	443.2 ± 42.8 b
	II	325.1 ± 23.3	308.5 ± 16.0 c
	III	501.4 ± 55.5	601.7 ± 27.7 a
9-121-4	I	430.7 ± 21.7 b	429.3 ± 26.1 b
	II	646.3 ± 51.3 a	836.7 ± 42.0 a
	III	510.0 ± 20.6 b	786.6 ± 61.0 a
9-121-5	I	214.3 ± 9.4 c	207.0 ± 11.6 b
	II	298.7 ± 20.0 b	386.5 ± 37.3 a
	III	368.4 ± 18.9 a	441.6 ± 44.8 a
11-110-2	I	239.9 ± 24.5 b	242.0 ± 15.0 b
	II	631.8 ± 32.9 a	708.8 ± 17.9 a
	III	652.9 ± 21.2 a	730.2 ± 34.4 a
Abrikosovaya	I	357.7 ± 30.0 b	394.7 ± 17.1 c
	II	626.7 ± 53.0 a	592.5 ± 12.6 b
	III	623.3 ± 58.5 a	741.9 ± 71.4 a
Arbat	I	305.9 ± 33.4	276.3 ± 18.9
	II	287.9 ± 32.9	388.8 ± 20.9
	III	270.1 ± 16.4	337.3 ± 36.9
Gusar	I	230.7 ± 21.8	260.6 ± 12.2
	II	314.0 ± 13.1	333.0 ± 18.0
	III	274.2 ± 28.8	346.6 ± 31.2
Karamelka	I	388.2 ± 20.2	330.7 ± 38.5
	II	336.9 ± 12.3	498.6 ± 42.8
	III	373.5 ± 16.6	426.0 ± 33.2
Himbo Top	I	570.5 ± 32.2	600.2 ± 64.0
	II	590.4 ± 28.8	598.9 ± 12.0
	III	651.7 ± 25.4	729.8 ± 36.8
Octavia	I	95.0 ± 11.6 b	95.4 ± 6.6 b
	II	174.1 ± 21.0 a	180.0 ± 7.6 a
	III	116.3 ± 8.5 b	202.3 ± 11.6 a
Polana	I	360.6 ± 19.0 a	307.6 ± 31.4 a
	II	197.2 ± 15.5 b	152.1 ± 8.0 b
	III	230.5 ± 14.2 b	339.4 ± 8.7 a
Polesie	I	88.4 ± 10.9 c	70.1 ± 5.4 c
	II	324.5 ± 21.4 a	392.6 ± 20.4 a
	III	239.6 ± 20.1 b	281.0 ± 25.1 b
Polka	I	469.6 ± 35.1	441.8 ± 30.8
	II	413.1 ± 39.8	554.5 ± 34.3
	III	482.6 ± 56.9	576.8 ± 41.5
Porana Rosa	I	431.9 ± 25.1	440.4 ± 40.8 b
	II	494.0 ± 31.2	738.4 ± 60.4 a
	III	378.8 ± 23.9	437.3 ± 48.3 b
Silvan	I	507.4 ± 46.7	569.2 ± 63.5 b
	II	523.3 ± 16.7	590.7 ± 21.1 b
	III	596.5 ± 46.5	752.5 ± 21.8 a

Results are expressed as mean ± SE ($n = 3$). Different letters represent statistically significant differences ($p < 0.05$).
